# Supplementary material for: The RhoGAP activity of CYK-4/MgcRacGAP functions non-canonically by promoting RhoA activation during cytokinesis
Source: eLife. 2015 Aug 7;4:e08898. doi: 10.7554/eLife.08898 (PMC4552957; doi:10.7554/eLife.08898)
Supplement: Supplementary file 1. — C. elegans strains used in this study. DOI: http://dx.doi.org/10.7554/eLife.08898.028 [file elife08898s001.pdf]

Supplementary Table 1. *C. elegans* strains used in this study.

Strain No.	Genotype
N2	*wild type*
MG685	*mgSi43[cyk-4::gfp::pie-1 3'utr, cb-unc-119(+)] II; unc-119(ed3) III*
MG695	*mgSi44[cyk-4(ΔC1)::gfp::pie-1 3'utr, cb-unc-119(+)] II; unc-119(ed3) III*
MG716	*mgSi16[cyk-4(r459a)::gfp::pie-1 3'utr, cb-unc-119(+)] II; unc-119(ed3) III*
MG733	*mgSi19[cyk-4(e448k)::gfp::pie-1 3'utr, cb-unc-119(+)] II; unc-119(ed3) III*
MG804	*mgSi33[cyk-4(k495e, r499e)::gfp::pie-1 3'utr, cb-unc-119(+)] II; unc-119(ed3) III*
MG822	*mgSi16[cyk-4(r459a)::gfp::pie-1 3'utr, cb-unc-119(+)] II; cyk-4(ok1034)/unc-64 III*
MG725	*mgSi43[cyk-4::gfp::pie-1 3'utr, cb-unc-119(+)] II; ltIs44[pAA173; pie-1p-mCherry::PH(PLC1delta1), cb-unc-119(+)] V*
MG726	*mgSi44[cyk-4(ΔC1)::gfp::pie-1 3'utr, cb-unc-119(+)] II; ltIs44[pAA173; pie-1p-mCherry::PH(PLC1delta1), cb-unc-119(+)] V*
MG729	*mgSi16[cyk-4(r459a)::gfp::pie-1 3'utr, cb-unc-119(+)] II; ltIs44[pAA173; pie-1p-mCherry::PH(PLC1delta1), cb-unc-119(+)] V*
MG815	*mgSi33[cyk-4(k495e, r499e)::gfp::pie-1 3'utr, cb-unc-119(+)] II; ltIs44[pAA173; pie-1p-mCherry::PH(PLC1delta1), cb-unc-119(+)] V*
MG730	*nop-1(it142) III; mgSi43[cyk-4::gfp::pie-1 3'utr, cb-unc-119(+)] II; ltIs44[pAA173; pie-1p-mCherry::PH(PLC1delta1), cb-unc-119(+)] V*
MG737	*nop-1(it142) III; mgSi16[cyk-4(r459a)::gfp::pie-1 3'utr, cb-unc-119(+)] II; ltIs44[pAA173; pie-1p-mCherry::PH(PLC1delta1), cb-unc-119(+)] V*
MG816	*nop-1(it142) III; mgSi33[cyk-4(k495e, r499e)::gfp::pie-1 3'utr, cb-unc-119(+)] II; ltIs44[pAA173; pie-1p-mCherry::PH(PLC1delta1), cb-unc-119(+)] V*
MG717	*ced-10(n1993) IV; mgSi16[cyk-4(r459a)::gfp::pie-1 3'utr, cb-unc-119(+)] II; ltIs44[pAA173; pie-1p-mCherry::PH(PLC1delta1), cb-unc-119(+)] V*
MG764	*mgSi43[cyk-4::gfp::pie-1 3'utr, cb-unc-119(+)] II; zwIS151[nmy-2::rfp, cb-unc-119(+)]*
MG766	*mgSi44[cyk-4(ΔC1)::gfp::pie-1 3'utr, cb-unc-119(+)] II; zwIS151[nmy-2::rfp, cb-unc-119(+)]*
MG765	*mgSi16[cyk-4(r459a)::gfp::pie-1 3'utr, cb-unc-119(+)] II; zwIS151[nmy-2::rfp, cb-unc-119(+)]*
MG817	*mgSi33[cyk-4(k495e, r499e)::gfp::pie-1 3'utr, cb-unc-119(+)] II; zwIS151[nmy-2::rfp, cb-unc-119(+)]*
EU1404	*cyk-4(or749ts) III*

Strain No.	Genotype
MG797	*ect-2(xs110) II; cyk-4(or749ts) III*
MG776	*ect-2(xs111) II; cyk-4(or749ts) III*
MG855	*ect-2(xs110 generated by CRISPR) II; cyk-4(or749ts) III*
MG863	*ect-2(xs110 generated by CRISPR) II*
MG887	*ect-2(xs111) II*
MG886	*cyk-4*^{R459A}*/unc-64 III*
MG903	*ect-2(xs110) II; cyk-4*^{R459A} *III*
MG904	*ect-2(xs111) II; cyk-4*^{R459A} *III*
MG873	*unc-4(e120) ect-2(xs110) II;*<br>*ltIs37[pAA64; pie-1p-mCHERRY::his-58, cb-unc-119 (+)] IV;*<br>*ltIs44[pAA173; pie-1p-mCherry::PH(PLC1delta1), cb-unc-119(+)] zuIs45 [nmy-2::NMY-2::GFP, cb-unc-119(+)] V*
MG512	*cyk-4(or749ts) nop-1(it142)III*
OD239	*cyk-4(or749)ts ltIs38[pAA1; pie-1p-GFP::PH(PLC1delta1), cb-unc-119 (+)] III; ltIs37[pAA64; pie-1p-mCHERRY::his-58; unc-119 (+)] IV*
MG582	*cyk-4(or749)ts ltIs38[pAA1; pie-1p-GFP::PH(PLC1delta1), cb-unc-119 (+)] III; ced-10(n1993) ltIs37[pAA64; pie-1p-mCHERRY::his-58; unc-119 (+)] IV*
MG503	*nop-1(it142) ltIs38[pAA1; pie-1p-GFP::PH(PLC1delta1), cb-unc-119 (+)] III; ced-10(n1993)IV*
MG936	*nop-1(it142)III; ced-10(n1993)IV; mgSi16[cyk-4(R459A)::gfp rRNAi, cb-unc-119(+)] II; ltIs44[pAA173; pie-1p-mCherry::PH(PLC1delta1), cb-unc-119(+)] V*
NG103	*mig-2(gm103) X*
