# Supplementary material for: The RhoGAP activity of CYK-4/MgcRacGAP functions non-canonically by promoting RhoA activation during cytokinesis
Source: eLife. 2015 Aug 7;4:e08898. doi: 10.7554/eLife.08898 (PMC4552957; doi:10.7554/eLife.08898)
Supplement: Supplementary file 2. — List of oligonucleotides used in this study. DOI: http://dx.doi.org/10.7554/eLife.08898.029 [file elife08898s002.pdf]

Supplementary Table 2. List of oligos used in this study.

	Oligo No.	Sequences
MosSCI	MG4199	GAAACGTGGCACGCCGGCGTGGACTAATGG
	MG4200	CCGCCTTGTATGGCCGGCTAGCGAATTCCA
	MG4276	CGAGCCTTCAGGGCAACCACAC
	MG4277	TGCCCTGAAGGCTCGTGGACTC
	MG4202	GGAACGGCGTAAATACCTTCCTGCGTGAG
	MG4201	TATTTACGCCGTTCCTGGGCAGGTTAGAACT
	MG4489	CGGGTCTTTAAGATCCTCTAGGAACCGCTCCAAGGTGTCTG TGATGACCTC
	MG4488	GAGGTCATCACAGACACCTTGGAGCGGTTCCTAGAGGATC TTAAAGACCCG
	MG4070	CGTGGTATTGGTCTCATTGCGATGTCACGAG
	MG4071	GAGACCAATACCACGCCCCAAGACGA
sgRNA	MG4735	CGTCCCAGACTACGCCTAAACTAGTGATAAATG
	MG4736	CAGGGTTATTGTCTCATGAGCGGATACATATTTG
	MG4773	**TGCTCGTCACGATGAATCAGG**CAAGACATCTCGCAATAGG
	MG4774	**CCTGATTCATCGTGACGAGCA**GTTTTAGAGCTAGAAATAGC AAGT
	MG4890	**TTCTAACCTGCCCAGGAACG**GTTTTAGAGCTAGAAATAGCA AGT
	MG4891	**CGTTCCTGGGCAGGTTAGAA**CAAGACATCTCGCAATAGG
oligonucleotide template (ODN)	MG4801	TTGTATGGTGCCTGATTCATCGTGACGAGCAAGATGGTGAC ATTGACACAGTCTTCGAAT
	MG4902	ACATTGACAGTTCTAACCTGCCCAGGAACGGCGTAAATACCT TCCTGCGTGAGTCCACGA

* Target sequences of sgRNA are highlighted.
